# External fixator versus elastic stable intramedullary nail for treatment of metaphyseal-diaphyseal junction fractures of the pediatric distal femur: a case-control study

**DOI:** 10.1186/s12891-024-07469-z

**Published:** 2024-05-18

**Authors:** Yuwei Wen, Qiang Wang, Baojian Song, Wei Feng, Danjiang Zhu

**Affiliations:** Department of Orthopaedics, Beijing Children’s Hospital, Capital Medical University, National Center for Children’s Health, No. 56, Nalishi Road, Beijing, 100045 China

**Keywords:** Elastic stable intramedullary nail, External fixator, Distal femur fractures, Children

## Abstract

**Background:**

Several methods have been used for the treatment of pediatric distal femoral fractures, such as elastic stable intramedullary nail (ESIN), external fixator (EF) and plate osteosynthesis, but there has been no consensus about the optimal method. The purpose of this study was to compare the clinical outcome between EF and ESIN techniques used in metaphyseal-diaphyseal junction (MDJ) fractures of the pediatric distal femur.

**Methods:**

We retrospectively analyzed operatively treated MDJ fractures of pediatric distal femur between January 2015 and January 2022. Patient charts were reviewed for demographics, injury and data of radiography. All of the patients were divided into EF and ESIN groups according to the operation techniques. Malalignment was defined as more than 5 degrees of angular deformity in either plane. Clinical outcomes were measured by Flynn scoring system.

**Results:**

Thirty-eight patients were included in this study, among which, 23 were treated with EF, and 15 with ESIN. The mean follow-up time was 18 months (12-24 months). At the final follow-up, all of the fractures were healed. Although there were no statistical differences between the two groups in demographic data, length of stay, estimated blood loss (EBL), rate of open reduction, time to fracture healing and Flynn score, the EF was superior to ESIN in operative time, fluoroscopic exposure and time to partial weight-bearing. The EF group had a significantly higher rate of skin irritation, while the ESIN had a significantly higher rate of malalignment.

**Conclusion:**

EF and ESIN are both effective methods in the treatment of MDJ fractures of the pediatric distal femur. ESIN is associated with lower rates of skin irritation. However, EF technique has the advantages of shorter operative time, reduced fluoroscopic exposure, and shorter time to partial weight-bearing, as well as lower incidence of malalignment.

**Level of evidence:**

Level III.

## Background

Femoral fracture is a common trauma in children, with an incidence of about 14-20/100,000 [1]. However, distal femoral fractures are rare injuries which account for 18% to 31% of all femoral fractures [2, 3]. The peak incidence is between 10 and 12 years of age and more common among males [4], and the most common mechanisms of injury are sports activities and motor vehicle accidents. Besides, it has been reported that distal femoral metaphyseal fractures in non-walking children caused by abuse account for 50% of all femoral fractures [5].

Most femoral fractures require inpatient care and treatment, and surgeons’ decisions made for the treatment of pediatric femoral fractures are affected by many factors, including age, weight, fracture pattern and associated injuries. Elastic stable intramedullary nail (ESIN), plate osteosynthesis and external fixator (EF) have been used in the treatment of femoral fractures. Controversy continues regarding the optimal treatment for these injuries, especially the proximal and distal femoral fractures. Spica cast with or without previous traction is described as an effective treatment method [1, 4, 6]. However, there are associated complications including compartment syndrome, skin compromise, and psycho-social strain on the patient’s family. Featuring easy postoperative care, fast fracture union and early independent mobilization, surgical treatment has been proven to be a reasonable choice [1]. ESIN is recommended for the treatment of femoral shaft fractures in school-age children because of minimal invasiveness, quick recovery and few complications. However, there is an increased rate of angulation deformity for fractures located at proximal and distal femur [7], and ESIN has the highest incidence of unplanned surgery due to loss of reduction [8]. Plate fixation can decrease the incidence of malunion and has superior stability in axial and torsional loading [9]. However, it is associated with extensive invasiveness and scarring, as well as complicated implant removal procedure. For distal femoral fractures in skeletally immature children, plate fixation is associated with the development of valgus deformity [10]. Whereas, EF has a number of advantages including convenient operation, stable fixation, less invasiveness, and not affecting the treatment of associated injuries. Therefore, EF was used for open fractures, multiple comminuted fractures, or as a temporary fixation of fractures. In recent years, EF has been applied to the treatment of distal femoral fractures and achieved good results [11, 12].

The purpose of this study was to compare the operation data and clinical outcomes between EF and ESIN techniques used in metaphyseal-diaphyseal junction (MDJ) fractures of distal femur in pediatric age group. We hypothesized that EF is superior to ESIN for the treatment for MDJ fracture of distal femur in pediatric age group.

## Methods

### Demographics

The medical records of children with femoral fractures in our hospital from January 2015 to January 2022 were retrospectively analyzed. The inclusion criteria were as follows: The midpoint of the fracture line was located on MDJ; the fixation method was EF or ESIN; the follow-up time ≥12 months; age between 3 and 12 years; weight <50kg. Exclusion criteria: Pathological fractures; combination with other fractures of lower limb; fractures at other location of femur (femoral neck, subtrochanteric, shaft, and metaphysis, etc.); previous lower limb surgery or fracture; cerebral palsy; incomplete clinical data.

**Fig. 1:**
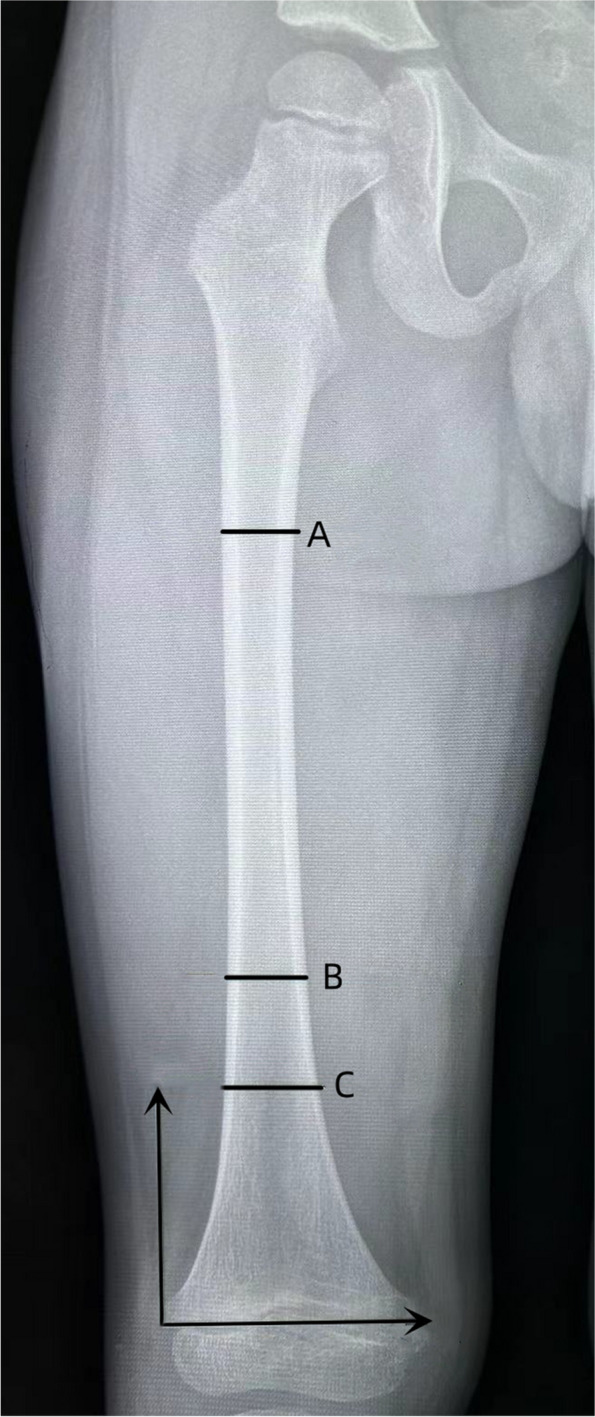
Definition of distal MDJ of femur. Line A and B represent 1/3 border of the proximal and distal femur, and line C represents the proximal border of metaphysis. We defined the MDJ as the femoral segment that lies completely at the proximal end of the distal 1/3 femur. It is bounded proximally by the junctional line between the middle and distal thirds of the femur and distally by the proximal end of the distal femoral metaphyseal region on the anteroposterior (AP) radiograph of the femur (Region line B-C)

According to the definition of metaphysis given by AO pediatric comprehensive classification of long bone fractures (PCCF) [13] and previous definition of MDJ [14], we defined MDJ as the femoral segment that lies completely at the proximal end of the distal 1/3 femur. It is bounded proximally by the junctional line between the middle and distal thirds of the femur and distally by the proximal end of the distal femoral metaphyseal region on the anteroposterior (AP) radiograph of the femur (Fig. 1. region B-C).

The following information was collected, including sex, age at the time of fracture, body mass index (BMI), affected side, mechanism of fracture, pattern of fracture, length of epiphysis, distance from midpoint of fracture line to epiphysis, time from injury to surgery, fixation mode, operation time, estimated blood loss (EBL), fluoroscopic exposure time and length of stay. During the follow-up, time to partial weight-bearing, time to fracture healing, re-displacement, angulation and complications were recorded.

Initial weight-bearing was performed with assistive devices, such as crutches and wheelchairs. In the current study, time to partial weight-bearing refers to the time until the children start to partially weight bearing. The fracture healing is defined as the appearance of bridging callus in three planes, as evidenced by the anteroposterior and lateral radiographs of the femur [11]. Malalignment was defined as more than 5 degrees of angular deformity in either plane. Flynn score [15] was used to evaluate the clinical outcomes. Low-energy injury mechanism includes ground-level fall, collision with another child, or fall from a height (lower than second floor), playground equipment, or a bicycle; while high-energy injury mechanism includes motor-vehicle collision, motor vehicle-pedestrian accident, or fall from the second or higher floor [6].

All of the children who met the inclusion criteria were divided into ESIN and EF groups according to the fixation method. Variables were compared between the two groups.

### Operative technique

All patients were placed supine on the operating table after general anesthesia.

**Fig. 2:**
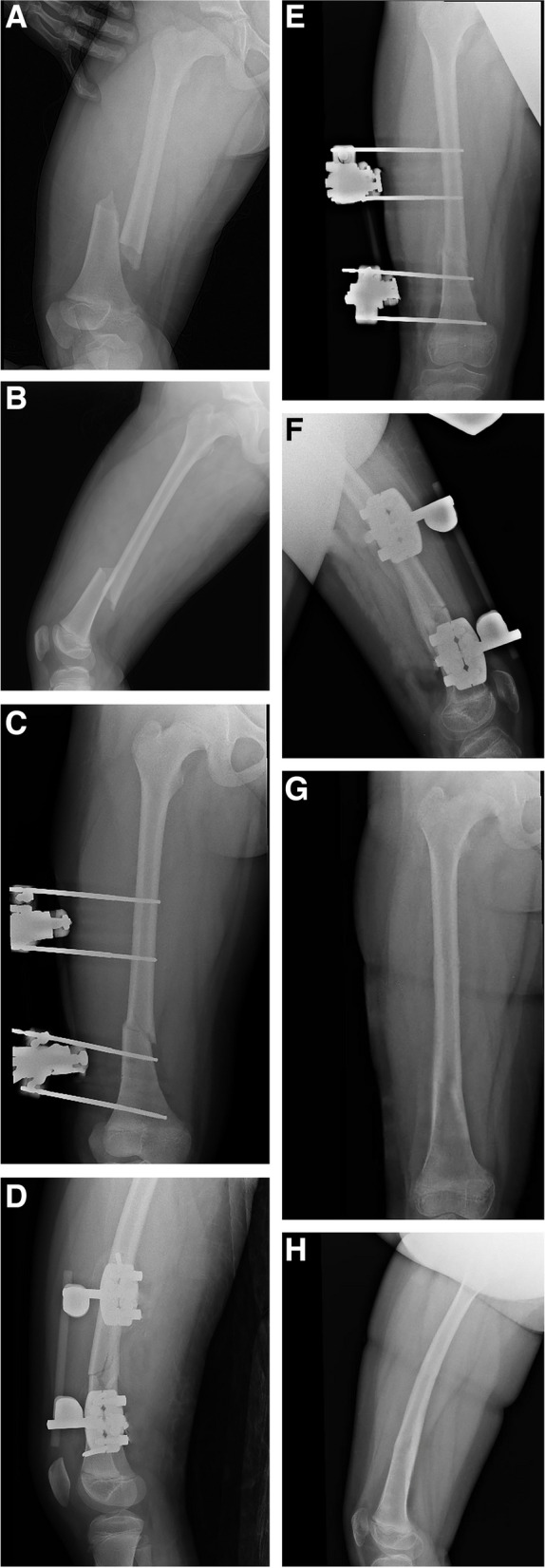
A 9-year-old girl with distal MDJ fracture caused by motor vehicle accident. Preoperative radiography showed complete displacement of the fracture with significant shortening (**A**, **B**); the fracture was treated with EF, and postoperative X-rays showed satisfactory reduction (**C**, **D**); with the fracture union after 3 months (**E**, **F**), the implant was removed under sedation, and the range of motion of the knee joint and lower limb function were normal at the final follow-up (**G**, **H**)

In EF group, the size of the Schanz pin was determined according to the age of the patients, generally ranging between 4.0 mm to 5.0 mm. We inserted the distal Schanz screws when the knee flexion was at least 90 degrees while avoiding the distal epiphyseal plate. The knee joint was actively mobilized for many times during the operation until no obvious muscle traction or entrapment was observed, so as to reduce postoperative pain and retain knee motion. Closed reduction was achieved in all patients with the assistance of joysticks (Fig.2).

**Fig. 3:**
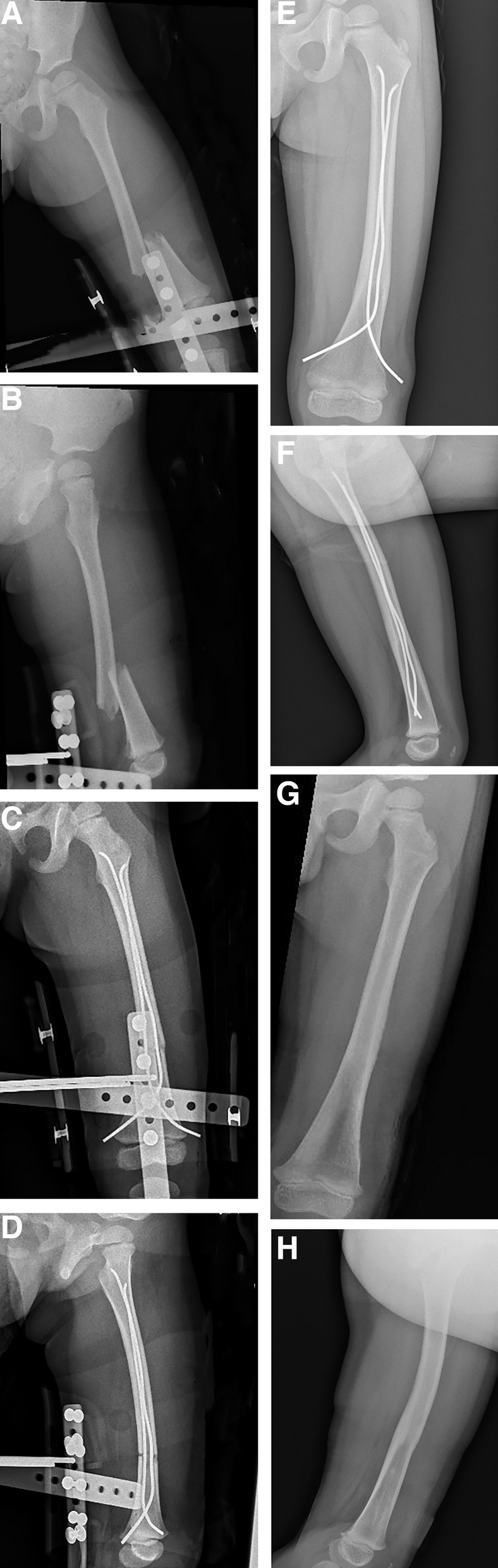
A 4-year-old girl with brace fixation in emergency department, with X-ray indicating MDJ fracture as a result of motor vehicle accident (**A**, **B**); the girl received ESIN treatment, and the intraoperative reduction was satisfactory (**C**, **D**). Additional brace was adopted to compensate for instability. With the fracture union after 6 months (**E**, **F**), the implant was removed under general anesthesia after 10, the knee activity and lower limb function were normal at the 13-month follow-up (**G**, **H**)

In ESIN group, the length of the proximal and distal fragment in MDJ fracture of distal femur and the diameter of the medullary cavity were measured before operation. Two nails of the same diameter were used to counter the imbalance caused by opposing bending forces, and the diameter of each nail was between 35% and 40% of the narrowest medullary canal. Previous studies found that retrograde technique offered greater resistance to bending in distal third fracture [16, 17]. In our study, ESIN was performed with retrograde technique. ESIN were prebent according to the measured length, so that the C-shaped vertex was as close as possible to the midpoint of the fracture to achieve the most stable support (Fig.3).

### Postoperative management

EF group: The Schanz pin site was dressed with medical iodine gauze to prevent infection and bleeding. If there was no exudation or bleeding 7 days after the operation, the tunnels were not covered again. The Schanz pin tunnels were kept clean and dry, and sterilized with ethyl alcohol twice a day. On the first day after the operation, passive functional exercise of the ankle and knee joints was initiated. For the first postoperative week, passive motion was applied within the range of 0 to 90 degrees. The passive motion was discontinued when full motion was obtained or when the patients were comfortable shifting to active motion activities. Weight-bearing exercises were performed 2-3 weeks after surgery. Full weight-bearing was allowed when adequate callus was formed in at least three planes. The EF was removed under sedation within 3 to 6 months.

ESIN group: The limbs were fixed with hip spica brace immediately after surgery. The patients began to be subject to quadriceps isometric contraction and ankle joint activity on the second day after the operation, but the knee joint was immobilized. The brace was removed and the weight-bearing exercises began upon the healing of fractures (evidenced by at least the appearance of bridging callus in three planes). The implants were removed under general anesthesia after fracture union.

Radiographs were reviewed for all patients. The distance from anterior superior iliac spine to medial malleolus was defined as leg length. Patients who showed any sign of limb-length discrepancy (LLD) underwent fluoroscopic examination involving the full length of both lower limbs.

### Statistical analysis

Statistical analysis was performed using standardized statistical software (Statistical Package for Social Science, version 23.0; SPSS, Inc., Chicago, IL). The Shapiro–Wilk test was used to verify the normal distribution of measurement data. Normally distributed variables were analyzed by t-test, and expressed as mean and standard deviation. Non-normally distributed data were analyzed by Wilcoxon rank sum test, and expressed as median (25^th^, 75^th^ interquartile range). The categorical data were analyzed by the Chi-square test. *P* values <0.05 were considered statistically significant.

## Results

**Fig. 4:**
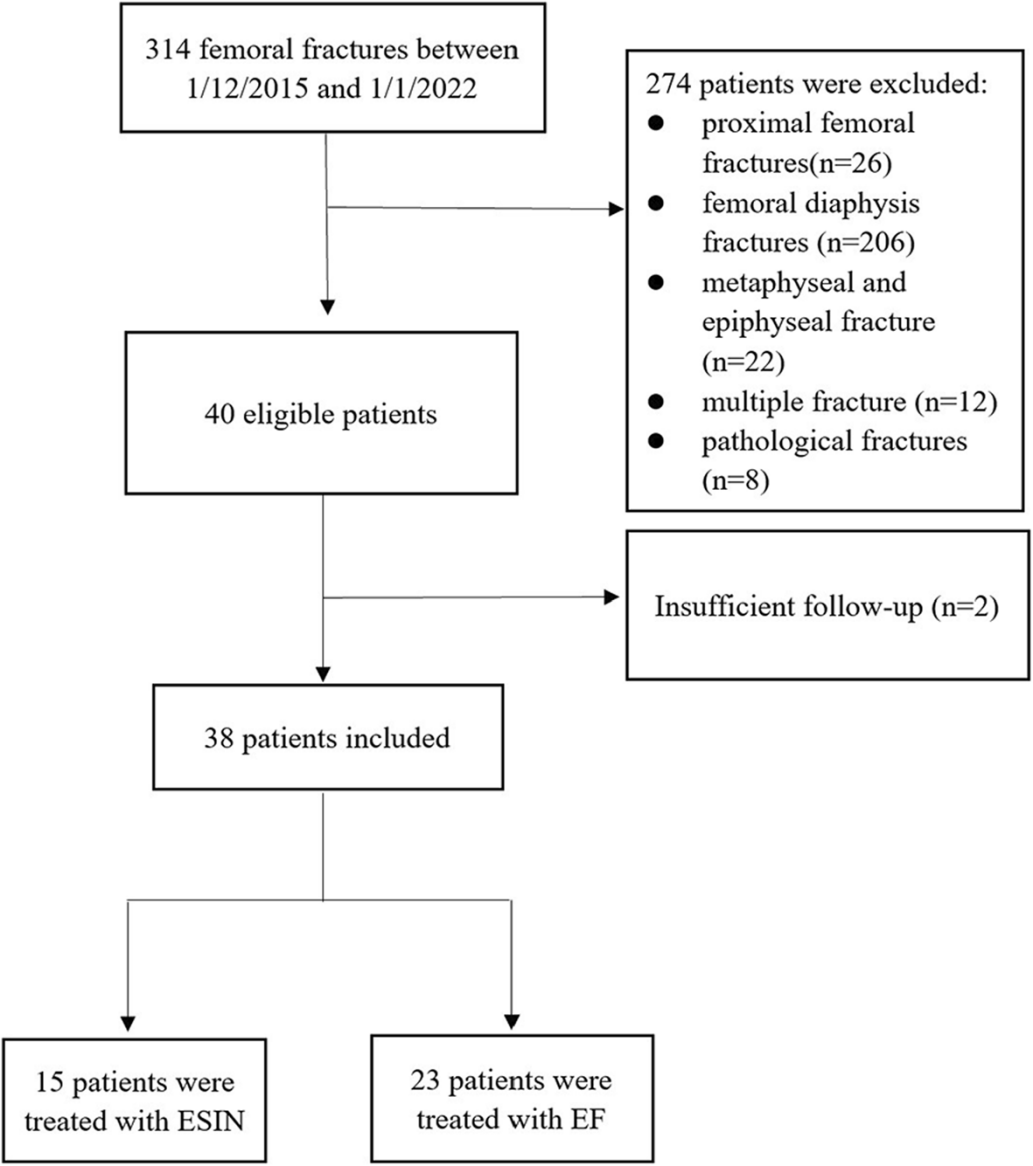
The patient flowchart

A total of 314 patients with femoral fracture were treated in our hospital from January 2015 to January 2022, and 38 children who met the inclusion criteria were included in this study (Fig 4). The mean age was 7.0 years (4.3-11.8 years), including 22 males and 16 females. The fractures were located on the left side in 19 patients and on the right in 19 patients. 26 fractures were caused by high energy mechanism and 12 by low energy mechanism. 15 patients were treated with ESIN, while 23 with EF.

All the fractures were healed at the final follow-up. In EF group, the mean time to partial weight-bearing was 2.4 weeks (2-3 weeks), the mean time to fracture union was 6.4 weeks (4-10 weeks), and the mean time to implant removal was 4.6 months (4-6 months). Eight patients with skin irritation were cured by wound disinfection and oral anti-inflammatory drugs. Three cases experienced LLD (the affected side is 0.8cm, 1.4cm, 1.5cm longer than the healthy side, respectively), and they could achieve normal gait by using insole without surgical intervention. Angulation was found in 2 cases (valgus 4 degrees and flexion 4 degrees). There was no malalignment, re-displacement, or refracture. The Flynn score was excellent in 16 cases and good in 7 cases, with an excellent and good rate of 100%.

In ESIN group, the mean time to partial weight-bearing and the mean time to fracture union were 5.6 weeks (4-9 weeks), and the mean time to implant removal was 7.9 months (6-12 months). One patient developed skin irritation; one unstable fracture was re-displaced after surgery, and the final follow-up showed a varus deformity of 13 degrees. At final follow-up, 4 patients experienced angulation, i.e., varus 13 degrees, valgus 4 degrees, flexion 9 and 8 degrees, respectively. Three of these angulations were defined as malalignment. Flynn score was excellent in 12 cases, good in 2 cases and poor in 1 case, with an excellent and good rate of 93.3%.

Demographic data were not statistically different between the two groups (Table 1). However, EF was superior to ESIN in respect to operative time, fluoroscopic exposure, and time to partial weight-bearing (Table 2).

**Table1:** Patient and injury demographics

Variables		ESIN	EF	t/x^2^	*p*
Age(y)		6.83±2.15	7.17±2.65	0.405	0.688
Side	Left	8(53.3%)	11(47.8%)	0.110	0.740
	Right	7(46.7%)	12(52.2%)		
Sex	Male	9(60%)	13(56.5%)	0.045	0.832
	Female	6(40%)	10(43.5%)		
Mechanism	High	11(73.3%)	15(65.2%)	0.277	0.599
	Low	4(26.7%)	8(34.8%)		
Length-stable	Stable	11(73.3%)	16(69.6%)	0.063	0.802
	Unstable	4(26.7%)	7(31.4%)		
BMI		19.60±4.11	20.16±6.85	0.285	0.777
Distance from fracture line to epiphyseal(cm)		7.08±1.57	6.92±1.57	-0.307	0.761
Epiphyseal length(cm)		5.18±0.86	5.50±1.27	0.733	0.469
Injury to surgery(d)		3.23±2.08	3.57±1.99	0.508	0.615

*ESIN* elastic stable intramedullary nail, *EF* external fixator, *BMI* body mass index

**Table 2:** Variables for clinical outcome

Variables	ESIN	EF	t/x^2^/z	*p*
Length of stay (d)	2.67±0.90	3.22±0.95	1.781	0.083
Operative time (min)	103.53±16.02	61.39±18.18	-7.309	<0.001
Fluoroscopy time (min)	2.93±0.99	1.60±0.50	-5.472	<0.001
Estimated blood loss (mL)	2.13±0.92	1.87±1.98	-0.481	0.634
Time to partial weight-bearing (w)	5(4,6)	2(2,3)	2.500	<0.001
Union time	5.60±1.64	6.43±1.72	1.485	0.146
Open reduction	2(13.3%)	0	3.237	0.072

*ESIN* elastic stable intramedullary nail, *EF* external fixator

In terms of complications, the LLD, re-displacement and Flynn score were not statistically different between the two techniques. Skin irritation was higher in EF group, while ESIN group reported significantly higher rate of malalignment (Table 3).

**Table3:** Complication and Flynn score

Variables		ESIN	EF	t/x^2^/z	*p*
malalignment		3 (20%)	0	4.994	0.025
Skin irritation		1 (6.7%)	8(30.4%)	3.971	0.046
Leg length discrepancy		0	3(13.0%)	2.142	0.145
Re-displacement		1 (6.7%)	0	1.575	0.210
Flynn score	Excellent	12 (80%)	16(69.6%)	2.798	0.248
	Satisfactory	2 (13.3%)	7(30.4%)		
	Poor	1 (6.7%)	0		

*ESIN* elastic stable intramedullary nail, *EF* external fixator, *LLD* limbs length discrepancy

## Discussion

The optimal treatment for pediatric femoral fractures is characterized by simple procedure, less trauma, early weight-bearing, maintenance of force lines of fractures until the formation of bridging callus, and minimal complications. In the current study, we found both EF and ESIN are good treatment options for distal MDJ femoral fractures, but EF is superior to ESIN due to shorter operative time, reduced fluoroscopic exposure, shorter time to weight-bearing, and lower incidence of malalignment.

Femoral fractures account for 2% of all pediatric fractures. MDJ is a special area of the distal femur, and in our study, MDJ fractures accounted for 12.1% of all femoral fractures, which was lower than the incidence of distal femoral fractures reported in the past. Salonen et al. [3] reported that distal femoral fractures occurred most frequently at less than 1 year old and between 12 and 16 years old, with the lowest incidence for children aged between 1 and 6 years old. In contrast, the average age of patients with distal MDJ fractures of femur in our study was 7 years old, mostly under 6 years old (69.7%). The male-female ratio was 1.4:1, not as six times more common in males as described by Duffy [4].

With the advantages of minimal invasiveness and few complications, ESIN has been considered as the most appealing option for femur shaft fractures [18–20]. However, Baghdadi [8] reported that ESIN treatment for femoral fractures in patients aged 5 years or older has the highest rate of unplanned reoperation, mainly due to complications. Malalignment was a major complication of ESIN, with the reported incidence ranging from 0 to 37 percent [7]. Previous studies have shown that the treatment of proximal and distal femoral fractures with ESIN may increase the risk of malalignment [7, 21]. In the current study, the malalignment of ESIN was 20%, consistent with previous studies. The reason is that when ESIN was used to treat distal femoral fractures, it was difficult to place the pre-bent vertex at the fracture to provide sufficient stability. Therefore, ESIN fixation of the distal femur requires additional brace or cast to compensate for the instability. In contrast, EF can achieve sufficient stability, thus lowering the risk of malalignment. Ricci et al. [7] reported that varus was the most common angulation, but the plane of the malalignment was nearly equally distributed. As revealed by our study, flexion was the most common angulation (3/6) and malalignment (2/3). The high incidence of flexion angulation may be caused by gastrocnemius traction and premature knee exercise.

To reduce angulation and re-displacement, long leg posterior splint immobilization for 3-4 weeks [22] or spica casting immobilization for 4-6 weeks [12] was adopted for patients who were treated by ESIN techniques. It was proved that ESIN was less effective in restoring full weight bearing (median: 6.8 weeks; range: 2.9-13.9 weeks) in complex fractures of the lower extremities [23]. In our study, spica brace was used in ESIN group for 4-6 weeks after surgery. The time to partial weight-bearing was 5.6 weeks (4-9 weeks). Previous studies reported that children with spica casts required care from their parents who took an average of 3 weeks off from work, and all of the children with the cast were forbidden to school [24]. The EF group does not require additional brace or cast to compensate for instability, thus avoiding complications associated with casts or braces. Furthermore, the time to weight-bearing of the EF (2.4 weeks) was significantly shorter than ESIN (5.6 weeks). Early weight-bearing is conducive to quick restoration of normal daily life, thus reducing the burden of family care and psychosocial strain on the patients’ family.

EF is associated with shorter operative time by approximately 0.7 hours compared to ESIN. It was previously reported that the operative time of ESIN was 0.9h [25] and EF was 0.75h[11]. In the current study, the operative time of ESIN was longer than previously reported. Retrograde ESIN provided more stability for distal femoral fractures [16, 17]. However, it was difficult to maintain reduction due to multiple muscles pulling the distal and proximal fractures, which prolonged the operative time. Two patients (2/15) from ESIN group required open reduction because of failed closed reduction. The EF technique was relatively simple, and joystick was used for assisted reduction, which reduced the operative time and the risk of open reduction. Longer operative time also predicts more intraoperative fluoroscopy. In the current study, ESIN fluoroscopy (2.9min) time was almost two times longer than EF (1.6min). This is consistent with what was reported in the prior literature [11].

Baghdadi et al. [8] reported that patients aged 5 years or older have the highest risk of unplanned reoperation, mainly related to the complications of flexible nails. Canavese et al. [22] found that patients have 8.3% risk of unplanned reoperation, mainly as a result of the loss of reduction. Submuscular plates have been used in treating distal femoral fractures to decrease malunion and achieve stable fixation [26, 27]. However, submuscular plates may damage epiphysis and cause growth disorders in children with skeletal immaturity, with the incidence of valgus reaching 30% among patients [10]. Allen et al. [26] hypothesized that plate and ESIN could achieve the same clinical results in the treatment of femoral fractures. The use of ESIN is associated with lower EBL, shorter operative time, and reduced cost compared with plate. Jin et al. concluded that EF is superior to plate in the treatment of distal femoral fractures, which shortens the healing and operative time, and reduces EBL [28]. In our study, there was no unplanned reoperation in either group. Only one patient reported re-displacement, with varus 13 degrees at the final follow-up visit, but the knee range of motion was normal, without reoperation.

It has been reported that EF is associated with a higher risk of skin irritation and refracture rate, and longer fracture union time [29–31]. In our study, the union time of the EF was 6.4 weeks, which was basically the same as ESIN (5.6 weeks). Li et al. [28] reported longer union time of EF, but it was still better than the plate. Although EF led to more skin irritation (34.7%) compared with ESIN, it was less than that (73.7%) reported by prior studies [11]. Neither of them required intravenous antibiotics and surgery, which was consistent with previous studies [11, 29]. In light of the fact that rich muscle coverage and muscle traction contribute to higher incidence of skin irritation, we recommend Schanz nails should be embedded when the knee flexion was higher than 90 degrees to reduce postoperative muscle stimulation. The refracture has an incidence rate of 2.1–21% [30]. Some studies have reported that premature fixator removal, open fracture, and open reduction were associated with refracture [31]. However, no refracture was reported in our study. In EF group, closed reduction was achieved in all patients with the assistance of joysticks, without open fracture. We recommend the EF should not be removed until adequate callus is formed in at least four visible cortices and no symptom is detected at full weight-bearing. We would not recommend aggressive strengthening exercises or any protocol that might overload the weakened bone for at least the first week after fixator removal.

LLD is also a common complication of femoral fracture. The factors related with overgrowth have been reported to be instability of fracture site, stimulation of growth plate, or disruption of periosteum [32]. If the stability at fracture site is related to overgrowth, the fixation method could be factors affecting the overgrowth after surgical treatment. There was no difference in the incidence of LLD between the two groups in our study, which was consistent with previous studies [11]. Thence, we think that the stimulation of growth plate and the disruption of periosteum were the primary reasons. In the EF group, 3 cases (13.0%) had LLD, none was greater than 2cm. The incidence of LLD is lower than plate [33], which might be due to the great damage to the periosteum during plate insertion and removal [34]. The patient’s age or sex may be related to overgrowth; however, there is controversy about the effect of these variables thereon [35, 36]. Implant removal is routinely offered at our institution. All children with ESIN require implant removal under general anesthesia, which causes anxiety among parents. In contrast, EF can be removed under sedation without secondary surgery, which is a special attraction to parents.

This study also has inherent limitations. This is a retrospective study, the assignment of patients to treatment groups is based on the preference of surgeons and parental requirements. Therefore, a selection bias may exist in the way that the patients were chosen for the procedures. The future research studies should consider a prospective design to validate the findings. The surgeries were performed by different doctors, who might adopt different surgical techniques. The number of cases differed between the two groups, which might lead to statistical errors. Besides, the overall number of cases is small, and studies with larger sample sizes are required.

## Conclusion

Both EF and ESIN are safe methods for the treatment of distal femoral MDJ in children. ESIN is associated with lower rate of skin irritation. However, EF has the advantages of reduced fluoroscopic exposure, shorter time to partial weight-bearing and operative time, as well as lower incidence of malalignment compared with ESIN. The use of EF avoids implant removal surgery, which is especially attractive for parents. Thus, the results of this study support the use of EF over ESIN in clinical scenarios where both options are equally feasible.

## Data Availability

No datasets were generated or analysed during the current study.

## Abbreviations

ESIN: Elastic stable intramedullary nailing
EF: External fixator
MDJ: Metaphyseal–diaphyseal junction
EBL: Estimated blood loss
BMI: Body mass index
LLD: Limb-length discrepancy
